# EAPD interim seminar and workshop in Brussels May 9 2015: Non-invasive caries treatment

**DOI:** 10.1007/s40368-015-0219-3

**Published:** 2016-02-10

**Authors:** C. van Loveren, W. van Palenstein Helderman

**Affiliations:** Department of Preventive Dentistry, Academic Centre for Dentistry, University of Amsterdam and VU University Amsterdam, ACTA, Gustav Mahlerlaan 3004, 1081 LA Amsterdam, The Netherlands; Professor Emeritus Department of Dentistry, Radboud University Nijmegen, Nijmegen, The Netherlands

**Keywords:** Non-invasive, Caries, Cavitated lesions, Non-cavitated lesions, Recall interval

## Abstract

**Aim:**

This was to collect information for the 9th European Academy of Paediatric Dentistry Interim Seminar and Workshops to discuss the state of art on non-invasive caries therapy to be used if possible to formulate clinical guidelines by European experts in paediatric dentistry

**Methods:**

Based on systematic reviews and additional papers were assessed for methods to prevent caries initiation and caries progression both in the state of pre-cavitation and cavitation without invasive technologies.

**Results:**

The use of fluoridated water, careful diligent daily use of fluoride toothpaste, fluoride varnishes, pit and fissure sealants and leak-proof restorative materials without excavation of caries are evidence based for caries prevention and for non-invasive treatment of pre-cavitated and cavitated caries. Other technologies are far less evidenced based and would not logically fit in guidelines for the non-invasive treatment of caries. Recent studies on cavitated lesions in the primary dentition demonstrate that thorough oral hygiene practices may arrest progression. This strategy depends heavily on the strategies in the dental surgery to change behaviour of children. An important aspect is for advice to be tailored at recall intervals to ensure compliance and to timely detect unnecessary and unwanted progression of the lesions.

**Conclusion:**

Non-invasive therapies have been proven to be effective for caries prevention and the management of pre-cavitated caries lesions. Non-invasive therapies can also be effective to arrest cavitated lesions but the success depends greatly on behavioural changes of patients to brush the lesions.

## Introduction

Dentistry often focuses on invasive and restorative treatments, maybe because it is most tangible and thereby rewarding. It is now recognised and accepted that surgical repair alone does not address the underlying aetiology of the disease (Ng et al. 2014). Unless the balance between de- and remineralization is altered, new and recurrent caries lesions are likely to occur. The dental literature indicates that children may be affected by an unfortunate circle of continuing dental caries. Young children who are not cooperative are sedated or treated under general anaesthesia. Despite receiving this costly treatment, children all too often develop new and recurrent caries (Ng et al. 2014). Recent literature suggests that for the primary dentition a successful rebalancing of risk and protective factors may completely halt or slow down the disease process, resulting in caries arrest even in cavitated lesions (Mijan et al. 2014; Santamaria et al. 2014). A pre-requisite is that the oral care provider guides parents and children this approach which, importantly, relies upon adequate care. When caries is a slow process, this approach is often effective. Gruythuysen et al. (2011) summarised the advantages of this approach, which is designated as non-restorative caries treatment (NRCT), or causal therapy, as follows: the self-efficacy of parents and child will be strengthened. Fear of dental treatment may reduce by the postponement or cancellation of restorative treatment. There is less burden for the child and possibly less referrals for GA treatment.

The fact that caries is still prevalent in many industrialised countries in spite of intensive preventive programs illustrates the limitation of these programs (Whelton et al. 2004). The question arises whether this limitation relates to inadequate number of measures (quantity), inadequate implementation of the measures (quality) or inadequate acceptance by patients and their parents (quality). In this, the studies of Hausen et al. (2000, 2007) are very illustrative. A intensive programme consisting of all known measures (Table 1) was not effective in high-risk children (Hausen et al. 2000). The same programme was shown to be effective [prevented fraction 44.3 % (95 % CI 30.2–56.4 %)] when the preventive programme was individually patient-centred, aimed at identifying and eliminating factors that had led to the presence of active caries. The programme included counselling sessions with emphasis on enhancing use of a children’s own knowledge and understanding in everyday life (Hausen et al. 2007). These studies showed that telling the patients what to do was not sufficient. Instead, healthcare providers should coach patients and parents about the factors that lead to and protect against dental disease and assist them in selecting self-management goals to improve their own and their children’s risk for disease (Ng et al. 2014).

**Table 1:** The preventive dental programmes used in the study of Hausen et al. (2000)

Basic programme (BP)	Intensive program (IP)
Duraphat 1×/year	Basic Programme
Fissure Sealants in deep fissures	+
Principles of good oral hygiene and diet were mentioned	Additional Duraphat varnish 1×/y
F-toothpaste 2×/day	All fissures sealed
No after-brush rinse	Intensive oral hygiene and dietary counselling. Xylitol chewing gum after meals
	Dental floss 3×/week
	CHX-gel 2×/y (for children with ≥10^6^ S.mutans/ml saliva)
	F-lozenges 4×/day

The transition from the traditional approach of oral health care providers relying on a surgical treatment model to a proactive disease management model coaching patients to improve selected self-management goals will not be easily achieved. Sbaraini et al. (2013) showed that adapting new preventive strategies to the existing routine of daily practice is a difficult process that slowly progresses against a range of barriers of practical, philosophical, and historical aspects of dental care. In particular, dentists spoke spontaneously about two deeply held ‘rules’ underpinning continued restorative treatment, which acted as barriers to provide preventive care: (1) dentists believed that some patients were too ‘unreliable’ to benefit from prevention; and (2) dentists believed that patients thought that only tangible restorative treatment offered ‘value for money’. Yet it was possible for dental practices to work against the normal teaching and implement prevention as their clinical norm.

The aim of the present article was to collect information for the 9th European Academy of Paediatric Dentistry Interim Seminar and Workshops which discussed the state of art on non-invasive caries therapy to be used if possible to develop clinical guidelines by European experts in paediatric dentistry (Kühnisch et al. 2016).

## Materials and methods

A literature search was made in April 2015 using PubMed, while The Cochrane Library was searched for systematic reviews. There were no language restrictions. The search terms used are given in Table 2. In total, 452 articles were screened by the authors by title and if necessary by summary. In the process, the authors noticed the large amount of systematic reviews on caries prevention which were recently qualitatively reviewed by Mejàre et al. (2015) against the AMSTAR criteria (Shea et al. 2007). Mejàre et al. (2015) concluded that the quality of evidence for the effectiveness of fluoride toothpaste for caries prevention was high, of other fluoride technologies low, of pit and fissure sealants moderate.The effectiveness of non-surgical methods to stop or reverse non-cavitated caries was uncertain while the quality of the evidence was very low.

No systematic reviews were found on the non-restorative caries management of cavitated lesions. It was noticed that very few studies on caries prevention reported baseline data making uncertain whether caries initiation or caries progression was affected. Recent studies focussed on individual approaches to care based on risk assessment making recall schemes important tools for caries management. In these systems, there was significant heterogeneity. For all these reasons, we decided to present the overview of methods for non-restorative caries management in a quantitative and narrative way.

**Table 2:** Overview of the search strategy used in Pubmed

		*N* of hits
#1	(“Child” [Mesh] OR children[tiab] OR “Adolescent”[Mesh] OR adolescent[tiab])	2,723,693
#2	Non-operative[All Fields] AND (“therapy”[Subheading] OR “therapy”[All Fields] OR “treatment”[All Fields] OR “therapeutics”[MeSH Terms] OR “therapeutics”[All Fields]) AND (“dental caries”[MeSH Terms] OR (“dental”[All Fields] AND “caries”[All Fields]) OR “dental caries”[All Fields] OR “caries”[All Fields])	32
#3	Non-restorative[All Fields] AND (“therapy”[Subheading] OR “therapy”[All Fields] OR “treatment”[All Fields] OR “therapeutics”[MeSH Terms] OR “therapeutics”[All Fields]) AND (“dental caries”[MeSH Terms] OR (“dental”[All Fields] AND “caries”[All Fields]) OR “dental caries”[All Fields] OR “caries”[All Fields])	15
#4	Therapy/Broad[filter] AND dental caries prevention[tiab]	254
#5	#4 AND #1	79
#6	Systematic[sb] AND dental caries prevention[tiab]	14
#7	Systematic[sb] AND (“dental caries”[MeSH Terms] OR (“dental”[All Fields] AND “caries”[All Fields]) OR “dental caries”[All Fields] OR “caries”[All Fields]) AND (“prevention and control”[Subheading] OR (“prevention”[All Fields] AND “control”[All Fields]) OR “prevention and control”[All Fields] OR “prevention”[All Fields])	413
#8	#7AND #1	207
#9	Systematic[sb] AND caries management[tiab]	27
#10	Therapy/Broad[filter] AND caries management[tiab]	117
#11	#2 OR #3 OR #5 OR #6 OR #8 OR #9 OR #10	452

### Technologies for caries prevention

There is evidence for the efficacy of fluoride technologies for caries prevention. The evidence for the effectiveness of pit and fissure sealants is less clear as is the evidence for chlorhexidine treatment and for dietary interventions. Pit and fissure sealant provide protection without reducing caries activity in the mouth (Heyduck et al. 2006). In Table 3, the systematic reviews are compiled for prevention of caries in primary and young permanent teeth.

**Table 3:** Overview of systematic reviews described by Mejàre et al. (2015) for the prevention of caries in primary and young permanent teeth

Technology	Outcome	Effect	References
Fluoride toothpaste	Caries prevention	PF 24 %, 95 % CI 21 to 28 %	Ammari et al. (2003); Twetman et al. (2003); Marinho et al. (2003)
	Supervised vs unsupervised (normally supervision by teachers)	PF 12 %, 95 %CI 0 to 21 %	Twetman et al. 2003
	Concentration: 440-450 vs 1000–1250 ppm F- 440–450 vs 1450–1500 ppm F-	PF 7 %, 95 % CI −9.5 to 24.8 % PF 14, 95 % CI −4.8 to 32.7 %	Walsh et al. (2010)
Fluoride varnish (risk of overestimation due to the limited number of studies)	Permanent dentition	PF 30 %, 95 % CI 0–69 %; PF 43 %, 95 % CI 30–57 %	Petersson et al. 2004 Marinho et al. (2013)
	Primary dentition	PF 37 % 95 % CI 24 to 51 %	Marinho et al. (2013)
Fluoride varnish application to children in school		No significant effect probably because the population with the greatest likelihood of decay did not consent to participate.	Hardman et al. 2007 (Cluster Randomised controlled trial)
Fluoride gel		PF 21 % 95 % CI 14 to 28 %	Marinho et al. (2004a)
Fluoride mouthrinse	Without background of F exposure	PF 29 % range 14 to 53 %	Twetman et al. (2004)
	With background of F exposure	PF 6 % range 0 to 30 %	Twetman et al. 2004
Fluoride mouthrinses, gels or varnishes used in combination with toothpaste		PF 10 % 95 %CI 2 % to 17 %	Marinho et al. (2004b)
Water fluoridation		–5.0 % to 64 % (median 14.6 %)	McDonagh et al. 2000
Resin fissure sealants	At 2 years of follow-up	OR 0.12, 95 % CI 0.07 to 0.19	Ahovuo-Saloranta et al. (2013)
	At 48 to 54 months of follow-up	OR 0.21, 95 % CI 0.16 to 0.28	Ahovuo-Saloranta et al. (2013)
	1st molars	RR 0.67 95 % CI 0.55 to 0.83	Mejàre et al. (2003)
	2nd molars, premolars and primary molars	Incomplete evidence for a caries-preventive effect	Mejàre et al. (2003)
Chlorhexidine		Evidence inconclusive	Twetman (2004), James et al. (2010) and Slot et al. (2011)

The most commonly used fluoride technology is fluoride toothpaste both in populations with and without fluoridated drinking water. The relatively short clinical trials on fluoride toothpaste, fluoride rinsing and various fluoride applications may have underestimated the effect of fluoride on caries when the exposure continues. The cariostatic effect of in water fluoridation studies with prolonged fluoride exposure resulted in a greater reduction in caries than shorter exposures (Groeneveld and Backer Dirks 1988; Fejerskov et al. 2015). Two main uncertainties of the use of fluoride toothpastes are the preventive effect in pre-school children related to the risk of fluorosis and the optimum ppm-value of fluoride in toothpastes intended for (high caries risk) children. In addition, to use related factors have the potential to significantly affect the effectiveness of toothpastes, e.g. the frequency of tooth brushing, the post-brushing rinsing behaviour, and the sideways use of a toothbrush and movement of the brush during the eruption of the (pre)molars. Studies on these topics are scarce and the results unequivocal (Carvalho et al. 1992; Sjögren et al. 1995; Machiulskiene et al. 2002; Braga et al. 2009; Abanto et al. 2015).

Studies on preventive technologies normally use cavitation as an outcome measure and do not describe the baseline conditions of the teeth. Therefore, as noted before, these studies do not prove at which stage of the caries process the technologies were effective. The question remains whether initiation or progression is prevented and whether the technologies are effective once the dentine is exposed to caries. Are these technologies appropriate for the management of precavitated and cavitated caries lesions?

### Management of precavitated caries lesions

In a systematic review in 2001, Bader et al. (2001) judged the evidence for the efficacy of any given method for arresting or reversing the progression of non-cavitated carious lesions to be insufficient for any specific type of intervention due to the small number of studies and the lack of statistical testing in most studies. A recent review (Tellez et al. 2013) confirmed, however, the potential of fluoride interventions (varnishes, gels, and toothpastes) to have benefit in decreasing the progression and incidence of non-cavitated carious lesions. Studies using xylitol, CHX, and CPP-ACP vehicles alone or in combination with fluoride therapy are very limited in number and in the majority of the cases did not show a statistically significant reduction. Sealants and resin infiltration studies point to a potential consistent benefit in slowing the progression or reversing non-cavitated carious lesions (Griffin et al. 2008; Tellez et al. 2013). Martignon et al. (2010) reported that the percent of the caries progression among approximal surfaces that had been sealed was lower than those assigned to a home-based flossing control after 12 months (test: 27 %, control: 51 %) and 2.5 years (test: 46 %, control: 71 %). A second study conducted by the same authors (Martignon et al. 2012) that evaluated infiltration treatment and fissure sealants (FS) versus placebo found significant differences between infiltration versus placebo (percentage of lesions showing progression 32 versus 70 %, respectively, *p* value: 0.001) and sealants versus placebo (percentage of lesions showing progression 41 versus 70 %, *p* value: 0.029), but no statistical difference between FS and infiltration after a 3-year period. In another study, Paris et al. (2010) reported a significant difference in the percentage of proximal lesions with progression of lesion depth between infiltration treatment versus placebo (test: 7 %, placebo: 37 %, *p* value: 0.021).

Braga et al. (2009) compared the effect of the cross tooth-brushing technique (CTT) in erupting first permanent molars, application of silver diamine fluoride (SDF), and glass-ionomer fissure sealant (GIC). After 3 and 6 months, SDF showed a significantly greater capacity for arresting caries lesions than CTT and GIC. At 18- and 30-month evaluations, the three groups were equally effective.

### Management of cavitated lesions

One way to treat cavitated lesions non-invasively is to place a leak-proof restoration without the caries having been removed by excavation. Examples of these are the ultraconservative caries treatment described by Mertz-Fairhurst et al. (1998) for the permanent dentition and the Hall technique for the primary dentition (Innes et al. 2011).

Another possibility is to fortify the dentine by the application of fluorides. One agent in particular, silver diamine fluoride (SDF: Ag(NH_3_)_2_F), has good support for its effectiveness, based on a 30-month prospective controlled clinical trial reported by Chu et al. (2002). The study involved 376 preschool Chinese children with caries in their maxillary primary anterior teeth. Subjects were sequentially assigned to one of the five treatment groups: excavation +38 % SDF applied every 12 months; SDF applied every 12 months; excavation +5 % NaF varnish applied every 3 months; 5 % NaF varnish applied every 3 months; water control. They found that annual application of SDF was more effective in arresting dentine caries than an application of fluoride varnish every three months. Furthermore, the removal of caries tissue did not improve the effectiveness of SDF or fluoride varnish to arrest dentine caries. SDF may blacken the teeth which of course would need to be balanced against its efficacy. The efficacy of SDF to arrest dentine caries has been confirmed in various clinical studies (Llodra et al. 2005; Zhi et al. 2012).

A recent report advocates non-restorative cavity treatment (NRCT) (Gruythuysen et al. 2011). The objective is to inhibit or halt the caries process in the cavity by thorough twice daily brushing the cavity with fluoride toothpaste. It is often necessary to enlarge the cavity by removing overhanging edges with a dental hatchet instrument or by slicing to render it more accessible for the toothbrush. The caries activity can be slowed down through improved plaque as well as removal on top of the caries lesions (Mijan et al. 2014; Santamaria et al. 2014). A prerequisite is that whoever guides parents and child to understand this approach of adequate self-care and continuous monitoring is indispensable.

Mijan et al. (2014) found no difference after a 3.5-year period in the cumulative survival rates of primary molars after three treatment modalities: with more conventional restorative treatment using silver amalgam, atraumatic restorative treatment and ultraconservative treatment protocol. In the latter group, medium to large cavities were, if necessary, enlarged with a dental hatchet and daily cleansed with toothpaste and toothbrush. During the 3.5-year trial, a trained dental assistant supervised the toothbrushing daily on schooldays and taught children how to perform the bucco-lingual toothbrushing technique on all non-restored cavities. The assistant was trained in detecting plaque. She repeated the brushing demonstration if a child’s teeth were not clean. Children were advised to clean their teeth during vacations as during the school terms.

Santamaria et al. (2014) compared three caries management options for occluso-proximal cavitated lesions in primary molars: conventional restorations (CR; complete caries removal and compomer restoration), Hall technique (HT; no caries removal, sealing in caries with preformed metal crowns), and non-restorative caries treatment (NRCT; no caries removal, opening up the cavity, teaching brushing and fluoride varnish (Duraphat) application). There were 148 children with a minimum follow-up period of 11 months. Twenty teeth were recorded as having at least 1 minor failure: NRCT, *n* = 8 (5 %); CR, *n* = 11 (7 %); HT, *n* = 1 (1 %) (*p* = 0.002). Nine (6 %) experienced at least 1 major failure: NRCT, *n* = 4 (2 %); CR, *n* = 5 (3 %); HT, *n* = 0 (0 %) (*p* = 0.002). Individual comparison of NRCT and CR showed no statistically significant difference in minor or major failures.

Ng et al. (2014) reported that a programme that relied on brushing with 1,000 ppm F toothpaste and applying a smear of 1,000 ppm stannous fluoride to the cavitated lesions in addition to the application of fluoride varnish at recall interval (see Table 6) was successful in preventing early childhood caries (ECC) in preschool children compared with an historical control group. A survival analysis performed at the time of new cavitation between the two groups found that the children in the programme had 62 % lower risk of new cavitation than the control patients at any given time during the three year experiment (Ng et al. 2012).

### Behavioural management

When carrying out the systematic reviews in paediatric dentistry, Mejàre et al. (2015) identified knowledge gaps in prevention and non-operative treatment of caries in primary and young permanent teeth. These findings are indicated in Table 4. This list should be prioritised and supplemented by behaviour management techniques. Dental health professionals are mindful of the relationship between psycho-social determinants of health and their patient’s dental status. However they still tend to employ approaches to health promotion and patient education that solely involve traditional transfer of knowledge and the giving of advice. Such an approach ignores accumulated knowledge concerns motivational and volitional factors relating to adaptive behaviour in prevention and does not use theories of behavioural change.

Two separate reviews by Gao et al. (2014) and Cascaes et al. (2014) examined a total of 26 randomised controlled trials to assess the effectiveness of motivational interviewing (MI) on oral health-related clinical and behavioural outcomes. The effectiveness of motivational interviewing was measured in comparison to giving conventional education. The design and delivery of the motivational interviewing intervention differed across studies, ranging from one to seven MI sessions, lasting between 5 and 90 min, being delivered by different healthcare professionals (with and without previous MI experience), administered on adults, adolescents and parents with young children. Follow-up times, after the intervention was delivered, ranged from 1 month to 2 years. In terms of outcomes, a variety of target behaviours and oral health outcomes were assessed using a number of clinical and self-report measures. Harrison et al. (2007) reported on studies that investigated clinical and behavioural outcome measures. They found some evidence of a positive MI effect in reducing dental caries in children by changing the behaviour of parents. This study appeared in both reviews and was rated as having good quality (Gao et al. 2014; Cascaes et al. 2014).

**Table 4:** Gaps in knowledge concerning prevention and non-operative treatment of caries in primary and young permanent teeth. From Mejàre et al. (2015)

Proper amount and level of ppm fluoride in tooth pastes for pre-school children related to the risk of fluorosis
Effect of toothpaste introduction by age, optimal brushing time and post-brushing behaviour
Additional effect of fluoride mouthrinse in high caries risk children/adolescents
Effect of fissure sealing of permanent molars in populations with low caries risk
Effect of fissure sealing of permanent molars with glass-ionomer cements
Effect of fissure sealing of permanent molars with composite resin-based FS compared with glass-ionomer cements
Effect of fissure sealing compared with fluoride varnish application
Effect of fluoride varnish in primary teeth
Effect of chlorhexidine
Effects of varying other agents and methods and effect of adding fluoride to food
Effects of information, professional programmes, routine dental examinations and counselling
Effect of non-operative methods to arrest or reverse non-cavitated caries lesions

In a systematic review on one-to-one interventions to change dietary behaviour undertaken in a dental setting (Harris et al. 2012), only one study involving children was identified (Hausen et al. 2007). In that study, the experimental group received an “individually designed patient-centred preventive programme aimed at identifying and eliminating factors which had led to the presence of active caries”. The individualised programme of prevention was delivered by dental hygienists trained in counselling, including understanding stages of change and different strategies for counselling. That approach specifically include the diet, with emphasis on identifying when during the course of the day snacking occurred, and involving emphasis on the importance of regular meals, the role of fermentable carbohydrates in the caries process, and the harmful effects of frequent snacking. When the dietary data in the multi-intervention study by Hausen et al. (2007) were analysed, only one (using xylitol products more than three times a day) of the seven dietary behaviours investigated showed that a significant change had occurred (Harris et al. 2012).

### All at once or step by step implementation

From the above, it is clear that the use of fluoride is the basis of caries prevention. When reviewing oral health promotion programmes, Kay and Locker (1998) concluded that only oral health promotion which brings about the use of fluoride is effective for reducing caries, while chairside oral health promotion had been shown to be effective more consistently than other methods of health promotion.

Fluoride can be used at home and additionally in the dental surgery. The first is relatively cheap and many patients prove that diligent use of fluoride toothpaste is sufficient to prevent the development of caries. The application of fluoride by dental professionals is expensive and to have an acceptable cost-effectiveness ratio based upon selection of patients is warranted. This will undoubtedly lead to false-negative findings. The same problem applies to the use of FS.

When viewing published protocols, two strategies of prevention emerge: those protocols that present a basic programme to everyone to which measures can be added and those protocols that contain all known preventive measures for everyone. There is no clear consensus in favour of one of the strategies in terms of effectiveness. A problem of summing measures on top of each other is that the added measure always has a lower degree of evidence than with a first choice one. But when measures are presented simultaneously, they will differ in the level of evidence and are presented as equally effective. Offering several measures at the same time may overburden patients leading to reduced compliance even for the measure with the highest level of evidence. If the strategy contains only the basic preventive measure, there is always an alternative to offer a more comprehensive programme. But this is not possible when all measures are presented at the same time. Another risk of presenting more measures at once is that it may lose credibility if patients do not adhere to them, but nevertheless remain caries-free.

It is important to realise why a certain programme is not working. Are the proposed measures not powerful enough or do they lack the necessary compliance of self-care management?

In the dental literature, more and more preventive programmes arise which emphasise the importance of self-care management over and above additive measures. Key factors in these strategies are assessment of risk factors and the use of self-management goals (SMG’s). The dental professional should help each patient to set realistic SMGs for which he or she is motivated to adhere to. The professional should possess adequate techniques for this such as motivational interviewing. Essential parts of these strategies are structured recall intervals based on the presence of caries risk factors and indicators.

### Preventive programmes with tailored recall intervals

The oral care providers in the small community of Nexø (9,000 inhabitants) on the island of Bornholm in Denmark, developed and implemented a special non-operative caries treatment programme (NOCTP) for children in 1987 (Carvalho et al. 1992). The treatment regimen was based on three principles dependent on individually assessed recalls: 1. Education of parents, children and adolescents for understanding dental caries as a localised disease, 2. Intensive training in home-based plaque control. 3. Early professional non-operative intervention (2 % NaF). Education of parents started when each child was 8 months old and attending the clinic for the first time. The parents were trained in home-based plaque control. The professional non-operative treatment comprised plaque removal by means of toothbrush or rubber cup and dental floss, and surface drying for visual examination for indications of caries progression. In case of further progression of dental caries more education and training in plaque removal is given and topical application of fluoride is considered. For the mixed and permanent dentition, the caries diagnosis was supported by radiographs if required. During the eruption of the first and second molars, special emphasis was given to brushing the occlusal surfaces by placing the brush transversal. There is a simple scheme to set the time between the recall visits based on diagnosis and compliance (Table 5).

**Table 5:** Overview of the system used in the Nexø-project to determine the individual recall interval

Criteria	Judgment	Score
Cooperation	Inadequate Good	2 points 1 point
Caries progression within the dentition	Yes No	2 points 1 point
Stage of eruption of permanent first/second molars	Partly erupted In Full occlusion	2 points 1 point
Occlusal surfaces of permanent first/second molars	Caries progression Caries free or arrested lesions	2 points 1 point

Recall interval based on the total number of points scored according to the criteria above			
Primary dentition		Mixed and permanent dentition	
4 points	1–3 months’ interval	8 points	1 months’ interval
3 points	4–8 months’ interval	7 points	2 months’ interval
2 points	8–12 months’ interval	6 points	3 months’ interval
		5 points	4 months’ interval
		4 points	6–12 months’ interval

The Nexo programme was successful (Carvalho et al. 1992; Ekstrand and Christiansen, 2005) and has been copied in other settings such as the Odder Municipal Dental Service in Denmark (Fejerskov et al. 2013), Moscow (Ekstrand et al. 2000) and the Netherlands (Vermaire et al. 2014). The interesting starting point of the programme is to use as few resources as possible. This has resulted in only the use of those measures with the highest level of supporting evidence. Vermaire et al. (2014) started the programme when the children were 6 years of age to prevent caries development in the first permanent molars.

In the literature, other systems have been described to determine tailored recall or disease management intervals. For example, Ng et al. (2012, 2014) reported a programme that relied on tailored disease management intervals based upon clinical findings as indicated in Table 6.

**Table 6:** Clinical findings determining the individual disease management recall interval (Ng et al. 2012, 2014)

Risk category	New clinical findings	Disease management return interval + fluoride varnish interval (months)
Low	(i) No disease indicators of caries (ii) Completely remineralised (arrested) carious lesions	6–12
Medium	(i) No disease indicators* but has risk factors** and/or inadequate protective factors*** (ii) Disease indicators present with some remineralisation	3–6
High	(i) Active caries (disease indicators present) (ii) No remineralisation occurring (iii) heavy plaque	1–3

* Examples of disease indicators including demineralisation, cavitated lesions, existing restorations, enamel defects, deep pits, and fissures

** Examples of risk factors including patient/maternal/family history of dental decay, plaque on teeth, and frequent snacks of sugars/cooked starch/sugared beverages

*** Examples of protective factors include fluoride exposure (topical and/or systemic) and xylitol

Abanto et al. (2015) reported on a preventive programme with tailored recall intervals that consisted of preventive strategies (oral hygiene instructions, dietary advice) and non-operative intervention for non-cavitated lesions with Duraphat for children under the age of 6 and APF-gel for older children (Table 7). If occlusal cavitated caries lesions were detected, those on the outer half of dentine were sealed with resin-based sealant with no previous removal of carious tissues. Deeper caries lesions, reaching the inner half of dentine, were not treated in the Prevention Clinic, but those patients were immediately referred to the Paediatric Clinic for timely treatment. Unfortunately, there was no comparison with a control group not receiving a programme with tailored recall intervals.

**Table 7:** Caries risk assessment and determination of recall intervals used by Abanto et al. (2015)

Classification	Group	Clinical conditions	Recall interval
Low risk	A	Absence of cavitated caries lesions or restored teeth, without dental plaque, without gingivitis and/or without active initial caries lesions	Every 12 months
Moderate risk	B	Presence of restored teeth. Absence of dental plaque, gingivitis and/or absence of active initial caries lesions	Every 12 months
	C	Presence of only inactive caries lesions associated with absence of dental plaque or gingivitis	Every 12 months
High risk	D	Presence of dental plaque, gingivitis and/or presence of active initial caries lesions associated with absence of cavitated caries lesion or restored teeth	Every 8 months
	E	Presence of one or more active cavitated caries lesions	Every 4 months

Evans and Dennison (2009) proposed a 10-step caries management system. Non-cavitated lesions are managed by home care measures to control plaque, principally by twice daily toothbrushing using fluoride toothpaste, thereby arresting lesion progression. In addition, the combination of professionally applied topical fluoride varnish and home use of fluoride toothpaste is necessary. This ensures that the natural repair process of remineralisation is accelerated and thus outweighs the effects of any remaining cariogenic challenge. Only cavitated lesions whose bases extend into dentine, or those so presumed to be cavitated in the absence of direct confirmation, are managed operatively. For less advanced pit/fissure lesions showing enamel breakdown, the most conservative and effective means of treatment is composite resin-based FS application (or a GIC sealant as an interim measure when there are concerns about moisture control), both to eliminate the accumulated plaque and to arrest further caries progression. The recall interval was tailored as indicated in Table 8. Unfortunately, the programme was not evaluated for children and adolescents with clinical outcome measures.

**Table 8:** Recall protocol for children and adolescents used by Evans and Dennison (2009)

Caries risk	Monitoring lesion activity and patient behaviour
Low	12 months after first visit Note: Oral hygiene review and coaching at each visit
At-risk… where evidence is: ICDAS II codes >1	3-monthly until lesion progression has arrested, i.e., evidence of (1) no extension of demineralisation or (2) that GIC sealant remains intact Note: Oral hygiene review and coaching at each visit
At-risk… where evidence is: Bitewing radiographs > C2* for primary teeth > C3** for permanent teeth	3-monthly for (1) F varnish and (2) oral hygiene monitoring until lesion progression has arrested and patient is reclassified as low risk Note: Oral hygiene review and coaching at each visit
At-risk… where only evidence is: Sites with Plaque Index = 3	One week following first visit to review and coach tooth brushing competence Then, 1 month later for same

* C2 Caries within the inner half of enamel

** C3 Caries involving the enamel dental junction

### Discussion

From the above, it is clear that the use of fluoridated water, the careful diligent daily use of fluoride toothpaste, the application of fluoride varnishes, the placing of FS and leak-proof restorative materials without excavation of caries are based upon evidence both for caries prevention and for non-invasive treatment of pre-cavitated and cavitated caries. Other technologies have far less supporting evidence and would not logically fit in guidelines for the non-restorative treatment of caries. Dietary advice takes a special place. It is clear and without dispute that the intake of sugars and fermentable carbohydrates is essential for caries to develop. Frequency of intake seems to be a more relevant determinant than the total amount. However, there are virtually no data on which frequency of intake is permissible when the teeth are twice a day carefully brushed with fluoride toothpaste. Some recommendations permit 7 times a day with main meals included, but this number seems to be related to convenience and not on scientific evidence.

Ecological studies reveal that a large part of many populations benefit sufficiently from the use of fluoride toothpaste while others do not. The reason for this is unanswered, but it could be argued that insufficient benefit results more from improper use of the products (improper compliance to the protocol) than from insufficient quality of them. It also emphasises whether this problem may be solved by adding products that will need proper use and compliance as well or by increasing the compliance to the original, simpler, more evidence-based protocol. To achieve increased compliance with a protocol, patients should develop self-management goals.

The key question then is whether increasing compliance to self-management goals can be acheived in a dental surgery. Is the dental professional willing and equipped to do so? The answer to this latter question may be crucial. If a dental professional is willing and knowledgeable to do so, then an effective disease management strategy based on self-care can be chosen for non-invasive caries treatment. If, however, a dental professional is not willing or prepared to do so, then a strategy based on office treatments, e.g. fluoride varnishes or sealants, is in line with expectations. The latter approach risks that a patient feels erroneously being protected neglecting his self-management.

### Suggested Protocols

**Fig. 1:**
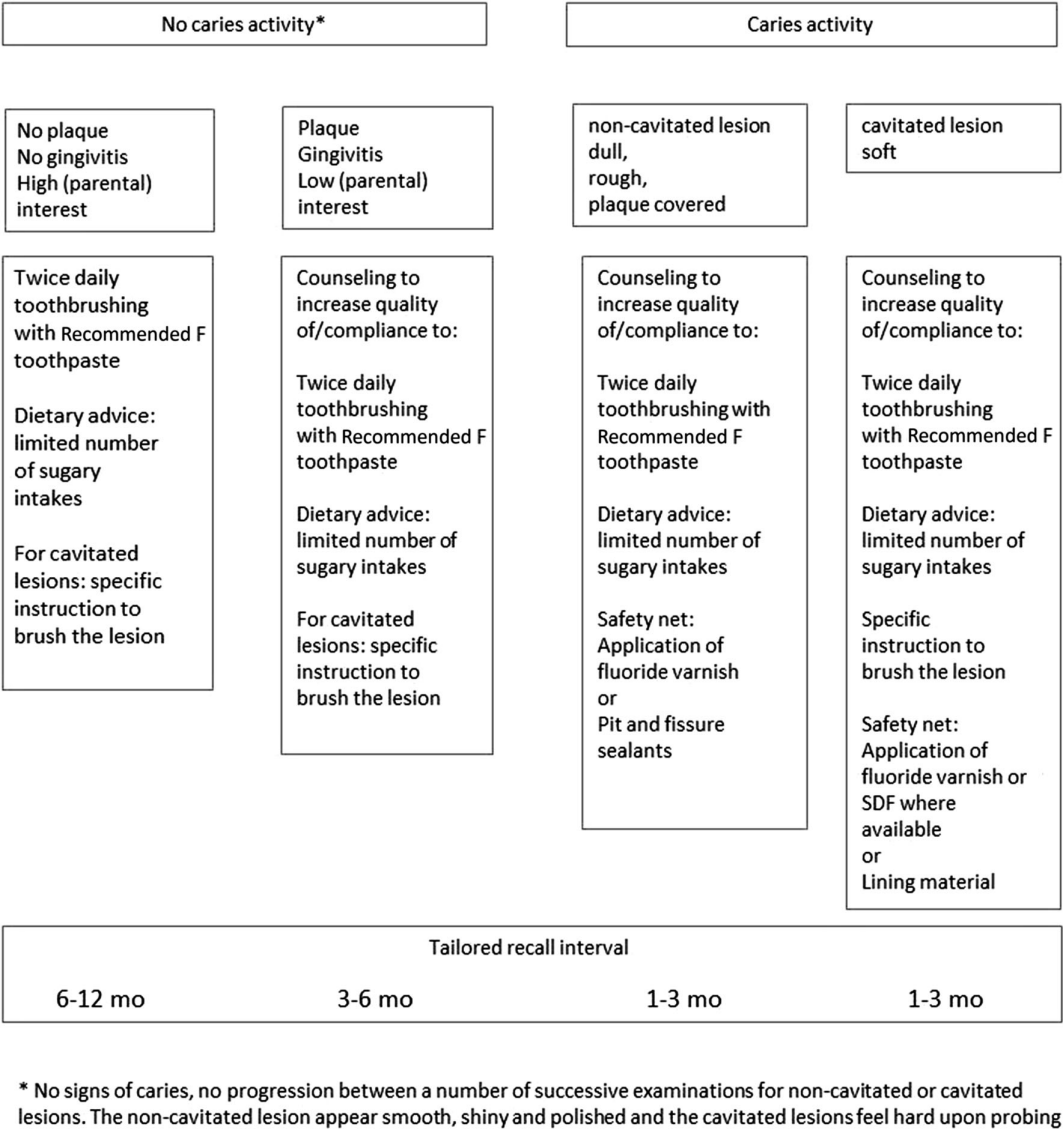
Flow diagram for non-invasive caries treatment

Based on these considerations, the following suggestions can be made for the non-invasive caries treatment (Fig. 1):

- The basic programme comprises twice daily tooth brushing with the recommended fluoride toothpaste and with limited number of sugary intakes.
- In case of inactive cavitated caries lesions instruction how to brush the lesions should be part of the basic programme.
- When there is no caries activity and there are no caries activity indicators present this should give sufficient protection and allow a recall interval of 6–12 months.
- No caries activity may be defined as no signs of caries or no signs of progression of non-cavitated or cavitated caries lesion between a number of successive examinations. The inactive non-cavitated lesions appear smooth, shiny and polished and the inactive cavitated lesions feel hard upon probing.
- Caries activity indicators are dull and whitish appearance, roughness, the presence of plaque, gingivitis and poor (parental) interest for oral health and its sustenance. Partial eruption may also be a caries activity indicator.
- When there is no caries activity, but caries indicators are present an effort should be made to achieve better compliance to the basic programme. A shorter recall interval may be required to achieve this.
- When there is caries activity, effort should be given to better compliance to the basic program. Pending the result of these efforts, fluoride varnish or pit and fissure or approximal sealants can be applied to non-cavitated active lesions. For active cavitated lesion, a lining material can be used to cover the surface and the protocol should be extended with a specific instruction to brush the lesion.
- Micro-, minimal- or invasive measures per se do not reduce caries activity.
- The decision to place the first restoration in a previously unrestored surface is a crucial event in the life of a tooth, because a permanent restoration, in the true sense of the term ‘permanent’, does not exist (Mjör et al. 2008).
